# The role of laterally transferred genes in adaptive evolution

**DOI:** 10.1186/1471-2148-7-S1-S8

**Published:** 2007-02-08

**Authors:** Pradeep Reddy Marri, Weilong Hao, G Brian Golding

**Affiliations:** 1 Department of Biology, McMaster University, Hamilton, Ontario L8S 4K1, Canada

## Abstract

**Background:**

Bacterial genomes develop new mechanisms to tide them over the imposing conditions they encounter during the course of their evolution. Acquisition of new genes by lateral gene transfer may be one of the dominant ways of adaptation in bacterial genome evolution. Lateral gene transfer provides the bacterial genome with a new set of genes that help it to explore and adapt to new ecological niches.

**Methods:**

A maximum likelihood analysis was done on the five sequenced corynebacterial genomes to model the rates of gene insertions/deletions at various depths of the phylogeny.

**Results:**

The study shows that most of the laterally acquired genes are transient and the inferred rates of gene movement are higher on the external branches of the phylogeny and decrease as the phylogenetic depth increases. The newly acquired genes are under relaxed selection and evolve faster than their older counterparts. Analysis of some of the functionally characterised LGTs in each species has indicated that they may have a possible adaptive role.

**Conclusion:**

The five Corynebacterial genomes sequenced to date have evolved by acquiring between 8 – 14% of their genomes by LGT and some of these genes may have a role in adaptation.

## Background

Bacterial genomes are constantly under pressure from the selective challenges of their surroundings. To overcome these hardships, bacterial genomes evolve *via *mechanisms in the form of genome modifications by gene loss [1-3], gene genesis by duplication, modifying existing genes by mutations [4,5] or acquisition of new genes by lateral gene transfer (LGT) [6-13]. Recent studies indicate that LGT has a larger role in bacterial evolution than previously anticipated [14-19], accounting for anywhere between 1.6 – 32.6% of the genes in each individual genome [20].

Gene content varies dramatically even among strains belonging to a single bacterial species [21-23]; variations mostly resulting from gene loss [1-3] and/or acquisition of new genes by LGT [6-13]. LGT plays a significant role in the evolution of bacterial genomes and provides them with a ready-to-use novel gene pool that helps them to adapt faster to their ever changing surroundings and foray into new ecological niches. Documented evidence shows that laterally acquired genes can transform an otherwise avirulent bacteria into a virulent form [24,25], protect pathogenic bacteria against antibiotics [26], increase the metabolic diversity of the recipient bacteria [12,27-29] or confer on it abilities to explore new challenging niches [30,31]. Keeping in mind the capability of LGTs to provide diverse adaptive features, we review some of the previous work done on lateral gene transfer in bacteria with an emphasis on the adaptive role of these laterally acquired genes. We also provide evidence about the transient nature of most of the laterally acquired genes based on a maximum likelihood modeling of the gene insertions/deletions at various stages during the evolution of the *Corynebacterium *species.

For the ease of discussion, we have classified the adaptive features into three major categories and will review each in turn: (1) Pathogenicity related features (2) Metabolic capabilities and (3) Survival under extreme environmental conditions.

### Pathogenicity related features

There are many documented instances of the acquisition of virulence determinants in bacteria by the process of LGT [22,25,32-38], selected examples are discussed below. The acquisition of a 35 kb *eaeA *locus encoding proteins responsible for attaching and effacing lesions has transformed an avirulent *Escherischia coli *strain into an enteropathogenic strain [39], whereas the acquisition of pathogenicity islands (PAIs) ranging from 70–150 kb and encoding virulence realted proteins resulted in uropathogenic strains [40,41]. Lawrence and Ochman [7] have identified that about 18% of the genome of *E.coli *MG1655 was acquired by LGT and this laterally acquired DNA has "conferred properties permitting *E.coli *to explore otherwise unreachable ecological niches".

The genome of *Salmonella enterica *has two laterally acquired pathogenicity islands, SPI-1 and SPI-2 encoding proteins that help in apoptosis, entry into non-phagocytic cells and systemic infection [42], whereas *Bacillus cereus *genome has three laterally acquired genomic islands BCGI-1, BCGI-2 and BCGI-3 with genes encoding proteins responsible for antibiotic resistance, ferric anguibactin transport system and lantibiotic biosynthesis leading to a better survival of *B. cereus *inside the host [43]. The highly pathogenic strains of *Yersinia pestis *have a 102 kb High Pathogenicity Island (HPI) that contains the *hms *locus encoding the capacity to store hemin, yersinibactin-pesticin receptor and an iron-regulated high molecular weight protein enabling an increased level of pathogenicity and survival in their hosts [44]. The case with the *cag *pathogenicity island in *Helicobacter pylori *is similar. This laterally acquired region encodes many antigenic determinants and virulence factors indicating its role in pathogenesis [45].

A comparison of the virulent and benign strains of *Dichelobacter nodosus*, a principal causative agent of the ovine footrot, revealed that the acquisition of *vap *and *vrl *regions encoding several virulence related genes has transformed an otherwise benign strain into a virulent strain [46,47]. Similarly, the virulent strains of *Vibrio cholerae *acquired a 45 kb PAI that includes the *tcp-acf *gene cluster involved in colonization and the *toxT *gene involved in the regulation of cholera toxin. This region, absent in the corresponding avirulent strains, plays a role in virulence and host adaptation [48].

This data indicates the ability of the laterally acquired genes to transform an otherwise non-pathogenic bacteria into a pathogenic bacteria. Interestingly, all the examples involve the acquisition of an adaptive trait, virulence, by acquiring large islands of linked genes.

### Metabolic adaptations

#### Primary metabolism

A recent study on the evolution of metabolic networks in *E. coli *by Pal et al [27] suggested that most of the laterally acquired genes have specific adaptive roles. Another study based on subtractive hybridisation by Espinosa and Kolter [49] has shown that the laterally acquired *gapC *gene has an adaptive role in the survival of *E. coli *in aquatic environments. Jahreis et al [50] demonstrated that the acquisition of the *CTscr94 *locus (consisting of *scr *genes that encode a phosphoenol-dependent phosphotransferase system involved in the sucrose fermentation pathway) broadens the metabolic versatility of *Salmonella senftenberg *and confers on it the capability to utilise sucrose as the sole carbon source. Sullivan and Ronson [28] have demonstrated that the laterally acquired 500 kb symbiosis island from *Mesorhizobium loti *ICMP3153 confers symbiotic ability to non-symbiotic *Mezorhizobium *strains and enables the bacterium to expand its genome to exploit new environmental niches. Similarly, the acquisition of the cyanobacterial nitrogen fixation (*nif*) genes by *Wolinella succinogenes *might enable it to survive in environments outside the host [29].

#### Degradation of xenobiotics

The ability to degrade xenobiotics is a relatively new physiological development in bacteria and several instances of the adaptability of the bacterial communities to xenobiotics have been attributed to the acquisition of genes related to xenobiotic degradation by LGT [12,51,52].

*Pseudomonas *sp. strain B13 has a genomic island of about 105 kb containing the chlorocatechol degradative (*clc*) genes that have the ability to degrade 1,2-dichlorobenzene (CB). Ravatn et al [53] have demonstrated that the acquisition of the B13 specific *clc *genes by *Pseudomonas putida *increases its metabolic diversity and results in a better adaptation to higher concentrations of CB. Dejonghe et al [54] have demonstrated that the transfer of 2,4-dichlorophenoxy acetic acid (2,4-D) degradation plasmids pEMT1 and pJP4 from *P. putida *UWC3 into the indigenous bacteria of two different horizons of 2,4-D contaminated soil enhanced the ability of the native bacteria to degrade 2,4-D, whereas de Lipthay et al [55] have demonstrated that lateral transfer of the gene (*tfdA*) encoding 2,4-dichlorophenoxyacetic acid dioxygenase into *Ralstonia eutropha *and other indigenous phenol degrading strains enhances the phenoxyacetic acid degradative capacity of the recipient strains in soil.

Poelarends et al [56] have indicated that the acquisition of the haloalkane dehalogenase gene (*dhaA*) was the key factor in the evolution of the haloalkane degradation ability of *Rhodococcus rhodochrous, Pseudomonas pavonaceae *and *Mycobacterium *sp. Similarly, the acquisition of the *pcpB *gene encoding pentalchlorophenol-4-monoxygenase by *Sphingomonas chlorophenolicum *confers on it the ability to degrade 2,3,4,6-tetrachlorophenol and survive under high concentrations of this chemical compound [57].

From the examples discussed above it is clear that some of the laterally acquired genes have the ability to increase the metabolic diversity of recipient bacteria enabling them to explore new ecological niches.

### Survival under extreme environmental conditions

The genome sequence of the radiotolerant bacteria *Deinococcus radiodurans *has revealed that topoisomerase IB (that plays a role in recombination and for which a knockout mutant is more sensitive to UV radiation) and RNA-binding protein (that is involved in the regulation of UV related damage repair) were acquired by LGT from eukaryotes [58]. *D. radiodurans *has also acquired the genes belonging to the late embryogenesis abundant (*lea*) family from plants [58]. These genes give resistance against dessication in plants and might provide *D. radiodurans *with additional resistance to UV radiation, since there is a positive correlation between resistance to dessication and radioresistance [58].

A comparative genomic analysis of the peizophilic strains of *Photobacterium profundum *has identified that several genes that are common to all the strains and responsible for high pressure adaptation are probably laterally acquired. Some of these genes are upregulated under high pressure conditions possibly suggesting their involvement in high pressure adaptation of *P. profundum *[30]. The genome sequence of *Colwellia psychrerythraea *has revealed that the psychrophilic lifestyle of this organism is due to the acquisition of a few genes by LGT in addition to amino acid content variations. Though LGT was not a primary player in the adaptation of this organism to this lifestyle, some specific lateral acquisitions including some of the cold shock proteins (possibly acquired from *Vibrio *sp. and *Shewanella onediensis*) and proteins responsible for the synthesis and degradation of complex high molecular weight organic compounds (acquired from *Ralstonia eutropha*) are certainly adaptive [31].

The genome sequence of *Halobacterium *indicates that most of the components of the electron transport chain including NADH dehydrogenase (*nuo*), menaquinone (*men*) and cytochrome oxidase (*cox*) are similar to the corresponding genes in *E.coli *and *D.radiodurans*. This suggests the transfer of genes involved in aerobic respiration from eubacteria into *Halobacterium *by LGT [59]. Another thermophilic archaea, *Picrophilus torridus *has acquired the genes that have the ability to degrade the organic acids such as acetic and propionic acid, superoxide dismutase, peroxiredoxin and alkyl hydroperoxide reductase to cope with oxidative stress along with the genes that encode the main components of the electron transport chain [60].

In contrast to pathogenic adaptations, these adaptations have largely been acquired by the LGT of a single gene. The above examples spanning different bacterial genomes clearly emphasize the adaptive role of LGTs. However, the examples discussed above are only the success stories of laterally acquired genes. Not all of the laterally acquired genes are adaptive. Of the many genes that are acquired, only a few genes that are adaptive are retained and fixed in a population [61]. There have been some studies suggesting that most of the laterally transferred genes are not useful and are hence reduced to pseudogenes [62] and studies that indicated the inability of LGTs to translate into functional proteins [63]. Given the examples above emphasizing the adaptive role of LGTs and counter-examples that suggest that most of the LGTs are not adaptive, it is important to explore the adaptive nature of LGTs and how frequently they are adaptive, if at all.

## Results

To answer the question on the adaptive role of LGTs, we have employed a maximum likelihood method to infer gene insertion/deletion rates at various stages during the evolution of five sequenced *Corynebacterium *genomes and have performed a systematic study on the species specific genes to further understand the role of the laterally acquired genes.

### Insertion and deletion rates

**Figure 1:**
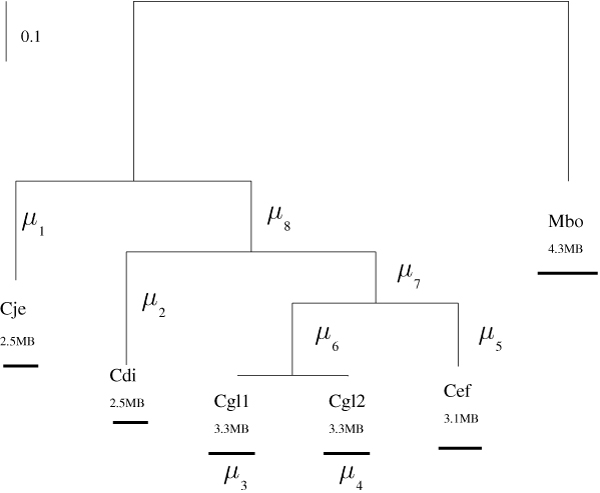
Bayesian tree obtained for the concatenated sequence of *fusA, gltS, infB, lysS, rplB, rpoB, secY, serS and *ychF genes. The abbreviations are: Cje (*Corynebacterium jeikeium*), Cdi (*C. diphtheriae*), Cgl_1 _(*C. glutamicum *ATCC 13032, NCBI accession No. NC_006958), Cgl_2 _(*C. glutamicum *ATCC 13032, NCBI accession No. NC_006958), Cef (*C. efficiens*), and Mbo (*Mycobacterium bovis*) as the outgroup. All branches are supported by posterior probability values of 100%. At least 80% of the trees for the commonly present single copy genes (534 genes in total) support branches 7 (with 82%) and 8 (with 86%), and trees for all genes support the remaining branches. **Model I**: Constant insertion/deletion rate on all branches. *μ *= *μ*_1 _= *μ*_2 _= *μ*_3 _= *μ*_4 _= *μ*_5 _= *μ*_6 _= *μ*_7 _= *μ*_8_. **Model II**: Branches separated into external and internal branches. *External*: *μ*_1 _= *μ*_2 _= *μ*_3 _= *μ*_4 _= *μ*_5 _= *μ*_6_; *Internal*: *μ*_7 _= *μ*_8 _**Model III**: Different insertion/deletion rate on each branch.

Gene insertions and deletions in closely related genomes can be inferred by looking at the phyletic patterns of gene presence or absence on a phylogenetic tree. Maximum parsimony and maximum likelihood methods have been successfully employed to understand the gene insertions/deletions [18,64-70]. The maximum likelihood analysis [70] was performed using the phylogeny derived from a concatenated DNA sequence of the em fusA, gltS, infB, lysS, rplB, rpoB, secY, serS and em ychF genes (Figure 1) and assuming that individual insertion and deletion events occur independently. The insertion/deletion rate of 1.18 (Ln*L *= -10875.4) was obtained using a model which assumes a single constant insertion/deletion rate (*μ*) on all branches (Model I). Branch lengths are measured relative to the estimated number of base substitutions, suggesting that there is one gene gained/lost for every nucleotide substitution. This rate is higher than that obtained from the study on *Bacillus *(0.51) [70], and close to that of *Streptococcus *(1.17) [71]. A second model, Model II, that assumes different insertion/deletion rates on external and internal branches (Figure 1) resulted in a higher rate (1.26) on external branches compared to the internal branches (1.06; Table 1) indicating that gene movement is greater on the external branches. The improvement in the likelihood although small is significant (*χ*^2 ^= Δ2 Ln*L *> 3.84 with d.f. = 1). A non-hierarchical model, assuming an irreversible gene loss, also shows a similar trend of a higher rate on external branches (1.33) over that on internal branches (0.60) (Table 1). The likelihood surface and the curvature of the likelihood surface are given as supplementary data (See Additional Files 9, 14 and 15).

A third model (Model III) considered a separate rate on each branch and also indicated that the external branches have an enhanced apparent insertion/deletion rate and that the rate of change varies greatly (Table 2). The large change in the likelihood from Model II to Model III with a small change in the number of parameters suggests that the fit of the model is significantly better. Interestingly, the rates on the external branches (*μ*_3_, *μ*_4_) leading to the two strains of *C. glutamicum *were higher compared to the other external branches. This is probably due to the larger genome size of *C. glutamicum *and smaller branch lengths. It is not due to the smaller number of genes differentiating these taxa since if these numbers of genes are halved, the rates remain comparable. Similarly, increased insertion/deletion rates on the branches leading to *C. glutamicum *strains (*μ*_6_) and *C. glutamicum *and *C. efficiens *(*μ*_7_) could be due to the fact that these represent the branches leading to species having larger genome sizes.

### Species specific genes

Species specific genes were divided into two categories based on BLAST and phylogenetic clustering; (1) Lateral gene transfers and (2) possible multiple deletion or lateral transfers (MDLT) (Table 3). The unique genes that cluster with BLAST homologues (at an E-value cutoff of 1.0 × 10^-10^) outside actinobacteria were considered as possible LGTs. Genes that were uniquely present in a *Corynebacterium *species but cluster with BLAST homologues from taxa within actinobacteria were considered as MDLT. The MDLT may possibly be instances of multiple deletions that are specifically retained by a single species or cases of intra-group gene transfers. LGTs reported here do not include the ORFan genes (as defined by [72]; genes with no hits to the NCBI **nr **database) of these species. As a result, the number of LGTs presented here will be a large under estimate of the actual number of LGTs in each of these species. The list of LGTs and their putative function along with the entire list of species specific genes can be found at [73].

The present study identified 215 genes specific to the *C. diphtheriae *genome. Of the 215 genes, 48 were possibly acquired by lateral gene transfer whereas the remaining 167 are MDLT. The functions of most of these genes are unknown (81 out of 215 encode hypothetical proteins), however, a comparison of the results with earlier studies on *C. diphtheriae *[74] indicate that many of these genes might encode proteins that are responsible for virulence related traits.

The study identified 283 *C. efficiens *specific genes, 225 are designated as MDLT while the remaining 58 are possibly acquired by LGT. A majority (194/283) of the species specific genes encode hypothetical proteins.

A comparison with three other corynebacterial genomes revealed the presence of 659 genes that are uniquely present in *C. glutamicum *species. These are the genes that are present in both the strains of *C. glutamicum *and absent in other corynebacterial species. This does not include the set of genes that are unique to a single strain of *C. glutamicum*. Of the 659 genes, 74 were acquired by LGT, 206 were identified as MDLT due to their presence in other actinobacterial taxa. There were 377 genes present only in the two strains of *C. glutamicum *and did not have hits in NCBI. Many (346/659) of the species specific genes encode hypothetical proteins. Unlike the other corynebacterial species, *C. glutamicum *has 58 membrane proteins, 16 regulatory proteins and 15 ABC transport related proteins (as classified in the annotation) indicating a greater metabolic diversity compared to the other species.

The study revealed the presence of 323 genes that are specific to *C. jeikeium*. Of the 323 genes, 292 are MDLT whereas the remaining 31 genes were possibly acquired by LGT. Again, a majority (179 of 323) of the species specific genes encode hypothetical proteins, however, some of the genes whose function is identified revealed that these genes were involved in the uptake of iron and manganese and hence might have a role in virulence and pathogenesis.

### Synonymous/non-synonymous changes and recent vs. ancient transfers

**Figure 2:**
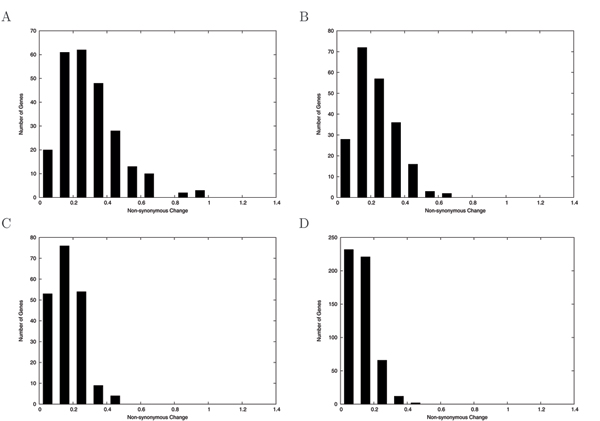
Non-synonymous change of different group-specific genes. Data is measured using the genes present in Cgl and Cef; genes present only in Cgl and Cef (A); genes present only in Cgl, Cef, and Cdi (B); genes present in only Cgl, Cef, Cdi, and Cje (C); genes present in all *Corynebacterium *taxa and Mbo (D).

**Figure 3:**
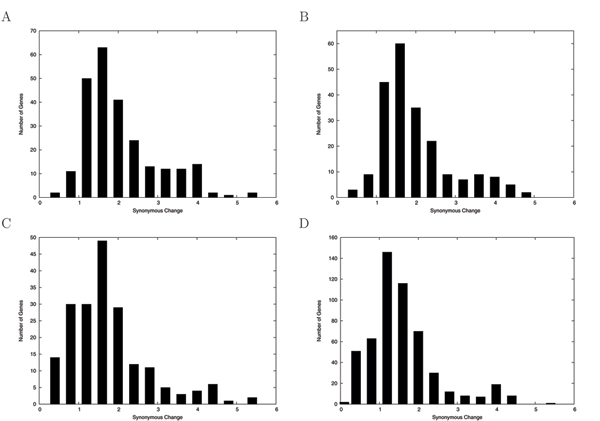
Synonymous change of different group-specific genes. Data is measured using the genes present in Cgl and Cef; genes present only in Cgl and Cef (A); genes present only in Cgl, Cef, and Cdi (B); genes present in only Cgl, Cef, Cdi, and Cje (C); genes present in all *Corynebacterium *taxa and Mbo (D).

**Figure 4:**
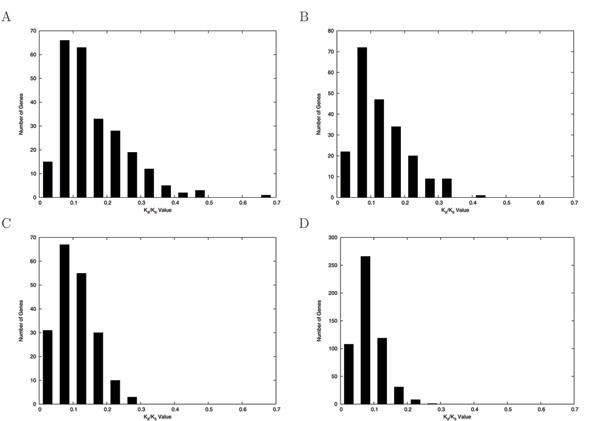
*K*_*a*_*/K*_*s *_ratio of different group-specific genes. Data is measured using the genes present in Cgl and Cef; genes present only in Cgl and Cef (A); genes present only in Cgl, Cef, and Cdi (B); genes present in only Cgl, Cef, Cdi, and Cje (C); genes present in all *Corynebacterium *taxa and Mbo (D).

The synonymous substitution rate (*K*_*s*_), non-synonymous substitution rate (*K*_*a*_) and their ratio (*K*_*a*_*/K*_*s*_) was measured for the orthologous genes between *C. glutamicum *and *C. efficiens*. The genes were divided into four groups based on the evolutionary time scale : genes present in *C. glutamicum *and *C. efficiens *only, genes present in *C. glutamicum*, *C. effciens *and *C. diphtheriae *only, genes present in all *Corynebacterium *species and absent in the outgroup and genes present in all the *Corynebacterium *species along with the outgroup. The genes present only in *C. glutamicum *and *C. efficiens *represent the putative most recently acquired genes while the genes present in all the species including the outgroup represent the most ancient genes. The results indicate that the genes that are inferred to have arisen recently via LGT in the phylogeny have more non-synonymous substitution changes than those that were transferred somewhat more anciently (Figure 2A versus 2B is *P *< 0.01 in a Wilcoxon rank test). As genes are resident in the species for longer periods the non-synonymous changes continue to go down (Figure 2B versus 2C is *P *< 0.01, Figure 2C versus 2D is *P *< 0.01). A similar trend was observed for *K*_*s *_and *K*_*a*_*/K*_*s *_where recently acquired genes had a higher rate of *K*_*s *_(Figure 3) and *K*_*a*_*/K*_*s *_(Figure 4) compared to the genes that have been resident longer. The analysis was repeated removing all the genes that did not have any homologs in non-*Corynebacterium *genomes, assuming that the genes present only in *C. glutamicum *and *C. efficiens*, and have no hits in the NCBI **nr **database are potential annotation artefacts. The distribution of *K*_*a*_, *K*_*s*_, and *K*_*a*_*/K*_*s *_values remains remarkably similar even after removing the uniquely present ORFs (see Additional Files 10, 11, 12) confirming the robustness of the result that recently acquired genes have higher rates of *K*_*a*_, *K*_*s*_, as well as a higher *K*_*a*_*/K*_*s *_ratio.

## Discussion

A maximum likelihood estimation of the gene insertion/deletion rate assuming a constant rate on all the branches was 1.18. This is higher than the inferred base substitution rates on the branches indicating that gene insertion/deletion plays a significant role in the evolution of these genomes. Genome rearrangement has been shown to have a minor role in the evolution of corynebacterial genomes [75] indicating that gene gain/loss might have a greater role in the evolution of *Corynebacterium *species. A model considering independent rates on each branch confirmed the enhanced rate of insertions/deletions on the external branches clearly showing a decrease in the rate with an increase in phylogenetic depth. The enhanced rate on the external branches of the phylogeny might indicate the transient nature of many of the laterally acquired genes. However, the observed difference between the rates on external and internal branches is not as dramatic as reported for the *Bacillus *group [70]. This may be due to the different phylogenetic relationship between the studies. If the transient nature of the LGTs holds true, one should expect lower rates of insertions/deletions on long external branches compared to short external branches. In fact, the rate of ins/del on short external branches in the *Bacillus cereus *group [70] is higher than the rate on external branches in this study. Furthermore, the rate estimation of insertion/deletion is robust for different cutoffs used for determining gene homologues. Different cutoffs (expect value *<*10^-20 ^with match length *> *85%, expect value *<*10^-10 ^with match length *> *70%, and expect value *<*10^-5 ^with match length *> *50%) show the similar trend that external branches tend to have higher rates of insertions/deletions than internal branches (see Additional Files 1, 2, 3, 4, 5, 6, 7).

In this study, the maximum likelihood estimation is based on a concatenated phylogeny of DNA sequences of nine genes with *Mycobacterium bovis *as the outgroup. The phylogenetic topology is supported by ribosomal RNA sequences (data not shown) and by the commonly present genes (Figure 1). The topology is robust regardless of the outgroup chosen from *Mycobacterium*, which is the closest phylogenetic neighbor of *Corynebacterium*. Furthermore, possible alternative topologies of the commonly present genes were evaluated using nine concatenated genes. The best supported alternative topology (see Additional File 13) is significantly worse than the topology used in the study. However, the maximum likelihood estimation based on the alternative topology still supports that recently acquired genes have high rates of ins/del (see Additional File 8). In this model, insertion rate and deletion rate were assumed to be equal. This assumption was based on the fairly constant genome sizes of the closely related taxa and also to ensure that in the long term, genome sizes would not tend to zero or infinity. In the study, all insertions/deletions were assumed to be independent and the rate of insertion/deletion was estimated from the gene phyletic pattern. Because of the difficulty of inferring insertion/deletion events after many genome rearrangements, the number of gene insertions/deletions rather than the actually insertion/deletion events was used in the maximum likelihood estimation. To overcome the simplistic assumptions in this study, more practical parameters, such as block ins/del rates, can be added to make the model more realistic in future studies.

The comparison of the synonymous (*K*_*s*_) and non-synonymous (*K_a_*) substitution rates of the genes that entered at various levels of the phylogeny indicate that the recently acquired genes evolve faster and have a higher proportion of synonymous as well as non-synonymous substitutions compared to their older counterparts (Figures 2,3). These results agree with earlier results indicating faster evolution of the recently acquired genes [70-72]. One of the possible reasons for faster rates of evolution could be that the newly acquired genes are required in the new habitat and are evolving faster to adapt to their new roles/habitat. Alternatively, some of these genes could be under relaxed functional constraint as they are non-functional in the new environment and might be evolving faster until they are deleted [70]. A more recent study has demostrated that low GC content causes seletive silencing of foreign DNA [76]. It was found that recently acquired genes tend to have lower GC content compared with ancient ones (see Additional File 16). This also supports the elevated evolutionary rates of recently acquired genes.

In this study, the phyletic patterns were derived primarily from genome annotation. As described previously in [70], non-annotated genes were picked up via a TBLASTN search and genes that are uniquely present in only one studied genome were removed from the study. Furthermore, a comparison was made by removing the ORFs only present in *C. glutamicum *and *C. efficiens *but not present in any other complete bacterial genomes (see Additional Files 10, 11, 12). The rates do not change remarkably after removing the Cgl-Cef group unique genes. This suggests that the fast rate of evolution of recently acquired genes is not an artifact of fast evolving non-gene ORFs in *C. glutamicum *and *C. efficiens*. A comparison of the corynebacterial genomes revealed that about 9 – 21% of the genes are specific to each species indicating that gene gain/gene loss has a major contribution in the evolution of these genomes. The results indicate that more than 10 – 35% of the species specific genes have been acquired by lateral gene transfer while the remaining are identified as possible MDLT (as inferred by their presence in other actinobacterial genomes).

The LGTs broadly fall into two categories; functionally characterised genes and hypothetical proteins. The analysis of the functionally characterised genes reveals that the pathogenic species *C. diphtheriae *and *C. jeikeium *have recruited genes that help in survival, host attachment and virulence. *C. diphtheriae *has acquired the genes responsible for iron transport [77,78] and siderophore biosynthesis that are directly involved in virulence. The acquisition of the genes encoding fimbrial subunits might indicate an adaptive mechanism whereby it helps in the attachment to the host cell surface [79], whereas the acquisition of the lantibiotic biosynthesis genes might help it to defend itself from other bacteria [80]. *C. jeikeium *has acquired genes that help in iron uptake. In addition, it has also acquired the genes necessary for uptake of manganese, another important requirement in pathogenesis [81]. The acquisition of the gene *cbpA *encoding a collagen binding protein might help in the bacterium-host interaction [82], whereas the presence of the genes *surA *and *surB*, encoding surface proteins, and the gene *acpA*, encoding an alkaline phosphatase, might help in virulence [83]. The acquisition of the neuraminidase encoding gene suggests that this might help *C. jeikeium *to prevent competition by other bacteria [84].

Unlike pathogenic species, the non-pathogenic species appear to have recruited many genes that are directly or indirectly involved in metabolic processes. *C. glutamicum *has acquired large numbers (53) of genes encoding membrane related proteins compared to the other species. Nishio et al [85] have indicated that many of the genes involved in amino acid biosynthesis are vertically inherited by *C. glutamicum *and only a few are acquired by lateral gene transfer. Our analysis identified only three genes (*brnE, mdh*2, *scrB*) that have been found to have a role in amino acid and vitamin biosynthesis [86]. The analysis of the LGTs in *C. efficiens *did not give many clues of their function as most of these genes encode hypothetical proteins.

We haven't considered in our study the ORFan genes [72] that account for about 10% of the genes in each of these species. Most of the recent studies on ORFans have confirmed that they are not a result of annotation errors but are in fact true genes, most of them being a part of genomic islands indicating their acquisition by LGT [23,72,87-89]. Given some of the examples reviewed here and based on the results obtained from this study on *Corynebacterium *species, it is compelling to suggest that at least some of these laterally acquired genes might have a role directly or indirectly in the adaptation of corynebacterial species.

## Conclusion

We demonstrate that 13 – 20% of the protein coding genes are specific to each *Corynebacterium *species and that these species have evolved mostly by LGT and gene loss. Maximum likelihood analysis indicates that there are more lateral transfers inferred at the tips of the phylogeny and that most of the lateral gene transfers are transient. Recently acquired genes evolve faster compared to their native counterparts. The faster rate of evolution of these recently acquired genes might reflect adaptation to new niches or might reflect rapid gene decay.

## Methods

### Genome sequences used

Five *Corynebacterium *genome sequences were obtained from NCBI [90] to carry out the analysis. They are *C. jeikeium *(Cje; [91]), *C. diphtheriae *(Cdi; [74]), *C. glutamicum *(NCBI accession No. NC_003450, Cgl1; [92]), *C. glutamicum *(NCBI accession No. NC_006958, Cgl2; [86]) and *C. efficiens *(Cef; [93]). *Mycobacterium bovis *(Mbo; [94]) was used as the outgroup.

### Maximum likelihood analysis

The evolutionary history of the *Corynebacterium *taxa was generated from the concatenated DNA sequences of *fusA, gltS, infB, lysS, rplB, rpoB, secY, serS and *ychF genes using Mr.Bayes ([95]; 200,000 generations sampled every 100 generations with a gamma distribution model and invariant class). The method to identify members of a gene family has been described in [66]. In brief, potential homologues were measured according to sequence similarities, and all paralogues in each genome were clustered as a single gene family and only one member was used for further analysis. The phyletic patterns (gene presence or absence in each genome) of all genes were used for the maximum likelihood analysis (see Additional File 1). The method to estimate likelihood has been described in [70]. Three different models, model I, II and III were used. Model I assumed a constant insertion/deletion rate on all the branches. In model II, the branches were separated into external and internal branches and rates were calculated separately for internal and external branches. In Model III the rates were calculated assuming each individual branch separately.

### Identification of species specific genes

The protein sequences from all the genomes were compared to each other using BLASTP [96], with an E-value cutoff set at 1.0 × 10^-20^ and an additional criteria of match length set at 85% of the query sequence. A set of genes present uniquely in a species and absent in the outgroup were considered as specific to that species. The species specific genes were checked to ensure that they are not simply a result of annotation errors. In the case of *C. glutamicum *with two intraspecific strains, all the genes that are present in both the strains and absent in other genomes including the outgroup were considered species specific. The genes present uniquely in a single strain were not considered. The species specific genes identified are sensitive to the parameters used in any study and to the number of taxa sampled.

#### Lateral gene transfer

The unique proteins of each species were compared to the NCBI **nr **database using BLASTP and with the expect value cutoff set at 1.0 × 10^-10 ^to identify homologs in other organisms. For each protein, the first 50 hits with an expect value less than 1.0 × 10^-10 ^were chosen for further analysis regarding possible lateral gene transfers. The complete protein sequences of these 50 hits were extracted from the GenBank database and a multiple alignment was done using ClustalW [97]. The multiple alignment was used to generate a phylogenetic tree using the neighbor joining (NJ) method [98] as implemented in PHYLIP [69]. The genes identified as LGTs by NJ trees were further confirmed by reconstructing phylogenetic trees using PROML (JTT + invariable class + *γ *distribution model) with rate paratmeter *α *calculated by Tree Puzzle [99].

### Synonymous/non-synonymous substitution rate

Synonymous changes and non-synonymous changes were measured by the PAML package [100]. The tree length of synonymous changes was calculated as the sum of the branch lengths for the taxa only within Cgl and Cef using the maximum likelihood method from the PAML package. Genes were categorised into four groups based on their presence/absence in different taxa (and hence on the inferred time period when the genes were transferred). The four groups are characterised by genes present in Cgl and Cef; genes present in Cgl, Cef, and Cdi; genes present in Cgl, Cef, Cdi, and Cje; and genes present in Mbo and all *Corynebacterium *taxa. Single copy protein sequences and their corresponding DNA sequences were extracted from the annotated genomes. Protein sequences were aligned using ClustalW [97], and nucleotide sequence alignments were created from the protein alignments by replacing each amino acid with its corresponding codon.

## Authors' contributions

PRM and WH contributed equally to this work. PRM, WH and GBG conceived the idea. WH performed the likelihood analysis and the measurement of *K*_*a*_/*K*_*s*_, PRM performed the LGT analysis. PRM, WH and GBG wrote the manuscript. All the authors have read and approved the manuscript.

## Supplementary Material

Additional File 9 Curvature of likelihood change in different models. Click here for file

Additional File 14 The likelihood value shows a continuous pattern of change as the insertion/deletion rate changes in the reversible gain/loss model. Click here for file

Additional File 15 The likelihood surface with different rates of insertion/deletion on external/internal branches in the reversible gain/loss model. Click here for file

Additional File 10 Non-synonymous change of the genes present in Cgl and Cef. A, genes present in Cgl and Cef; B, genes present in Cgl and Cef and having matches in the database. Genes show similar rates of evolution by only looking at the genes having matches elsewhere the genome database. Click here for file

Additional File 11 Synonymous change of the genes present in Cgl and Cef. A, genes present in Cgl and Cef; B, genes present in Cgl and Cef and having matches in the database. Genes show similar rates of evolution by only looking at the genes having matches elsewhere the genome database. Click here for file

Additional File 12 *K*_*a*_*/K*_*s *_ratio of the genes present in Cgl and Cef. A, genes present in Cgl and Cef; B, genes present in Cgl and Cef and having matches in the database. Genes show similar rates of evolution by only looking at the genes having matches elsewhere the genome database. Click here for file

Additional File 1 The phyletic patterns of the *Corynebacterium *taxa. (cut-off: expect value less than 10^-20 ^and match length over 85%) Click here for file

Additional File 2 The phyletic patterns of the *Corynebacterium *taxa (cut-off: expect value less than 10^-10 ^and match length over 70%) Click here for file

Additional File 3 The phyletic patterns of the *Corynebacterium *taxa (cut-off: expect value less than 10^-05 ^and match length over 50%) Click here for file

Additional File 4 Insertion/deletion rates inferred from the maximum likelihood analysis assuming different rates for external and internal branches (cut-off: expect value less than 10^-10 ^and *> *70% match length) Click here for file

Additional File 5 Insertion/deletion rates inferred from the maximum likelihood analysis assuming different rates on each branch of the phylogeny (cut-off: expect value less than 10^-10 ^and *> *70% match length) Click here for file

Additional File 6 Insertion/deletion rates inferred from the maximum likelihood analysis assuming different rates for external and internal branches (cut-off: expect value less than 10^-05 ^and *> *50% match length) Click here for file

Additional File 7 Insertion/deletion rates inferred from the maximum likelihood analysis assuming different rates on each branch of the phylogeny (cut-off: expect value less than 10^-05 ^and *> *50% match length) Click here for file

Additional File 13 An alternative topology of the concatenated genes. The insertion/deletion rates on external branches and internal branches were studied. Click here for file

Additional File 8 Insertion/deletion rates inferred from the maximum likelihood analysis assuming different rates for external and internal branches. Likelihood was estimated using the alternative topology and a cutoff with expect value less than 10^-20 ^and *> *85% match length. Click here for file

Additional File 16 Decreased GC content of recently acquired genes. Click here for file

## Figures and Tables

**Table 1:** Insertion/deletion rates inferred from the maximum likelihood analysis assuming different rates for external and internal branches.

Rate	Reversible		Deleted once^*a*^	
	MLE	Ln*L*	MLE	Ln*L*
constant *μ*	1.18	-10875.4	1.08	-10991.8

*μ*_1 _= *μ*_2 _= *μ*_3 _= *μ*_4 _= *μ*_5 _= *μ*_6 _(external)	1.26	-10873.0	1.33	-10958.3
*μ*_7 _= *μ*_8 _(internal)	1.06		0.60	

^*a*^Genes can not be regained after deletion.

**Table 2:** Insertion/deletion rates inferred from the maximum likelihood analysis assuming different rates on each branch of the phylogeny.

Rate	Reversible		Deleted once^*a*^	
	MLE	Ln*L*	MLE	Ln*L*
constant *μ*	1.18	-10875.4	1.08	-10991.8

*μ*_1_	1.37	-10578.0	1.29	-10618.8
*μ*_2_	0.46		0.48	
*μ*_3_	4.60		4.87	
*μ*_4_	27.42		32.82	
*μ*_5_	0.98		1.06	
*μ*_6_	2.25		2.20	
*μ*_7_	2.49		2.13	
*μ*_8_	0.001		0.008	

^*a*^Genes can not be regained after deletion.

**Table 3:** Classification of unique genes in Corynebacterium species.

**Genome**	**Total Genes**	**Species specific genes (Hypothetical Proteins)**	**LGTs**	**MDLT**
*C. diphtheriae*	2272	215 (81)	48	167
*C. efficiens*	2950	283 (194)	58	225
*C. glutamicum*	3057	659 *(346)	74	206
*C. jeikeium*	2137	323 (179)	31	292

Numbers based on an E value cutoff of 1.0 × 10^-10 ^with no length cutoff.

*377 genes present in *C. glutamicum *strains only
