# Supplementary material for: The role of laterally transferred genes in adaptive evolution
Source: BMC Evol Biol. 2007 Feb 8;7(Suppl 1):S8. doi: 10.1186/1471-2148-7-S1-S8 (PMC1796617; doi:10.1186/1471-2148-7-S1-S8)
Supplement: Additional File 14 — The likelihood value shows a continuous pattern of change as the insertion/deletion rate changes in the reversible gain/loss model. [file 1471-2148-7-S1-S8-S14.pdf]

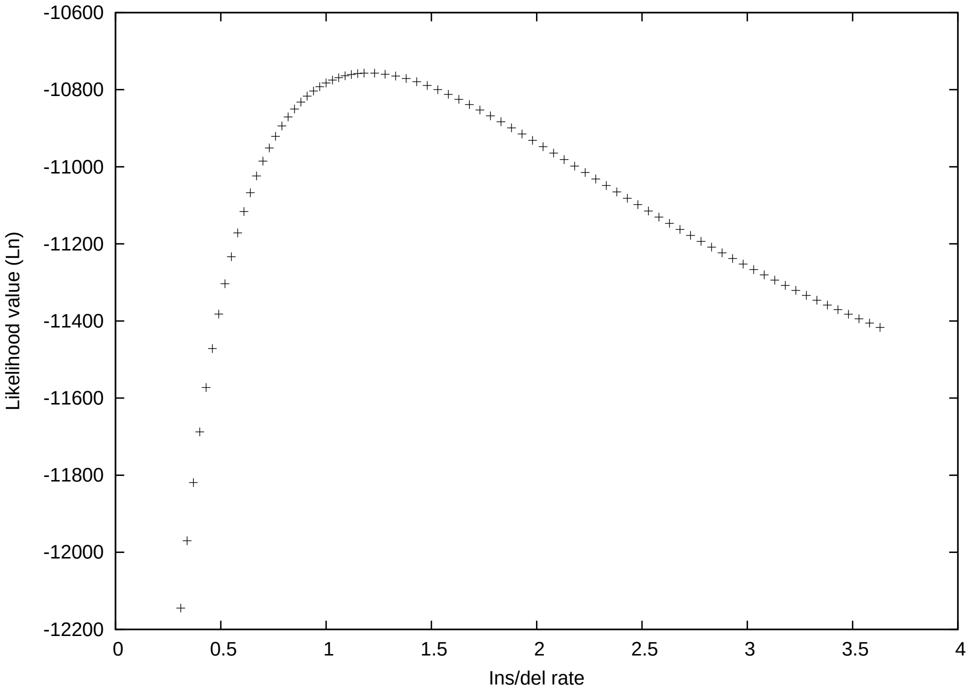


Figure S.5: The likelihood value shows a continuous pattern of change as the insertion/deletion rate changes in the reversible gain/loss model.
