# Supplementary material for: The role of laterally transferred genes in adaptive evolution
Source: BMC Evol Biol. 2007 Feb 8;7(Suppl 1):S8. doi: 10.1186/1471-2148-7-S1-S8 (PMC1796617; doi:10.1186/1471-2148-7-S1-S8)
Supplement: Additional File 11 — Synonymous change of the genes present in Cgl and Cef. A, genes present in Cgl and Cef; B, genes present in Cgl and Cef and having matches in the database. Genes show similar rates of evolution by only looking at the genes having matches elsewhere the genome database. [file 1471-2148-7-S1-S8-S11.pdf]

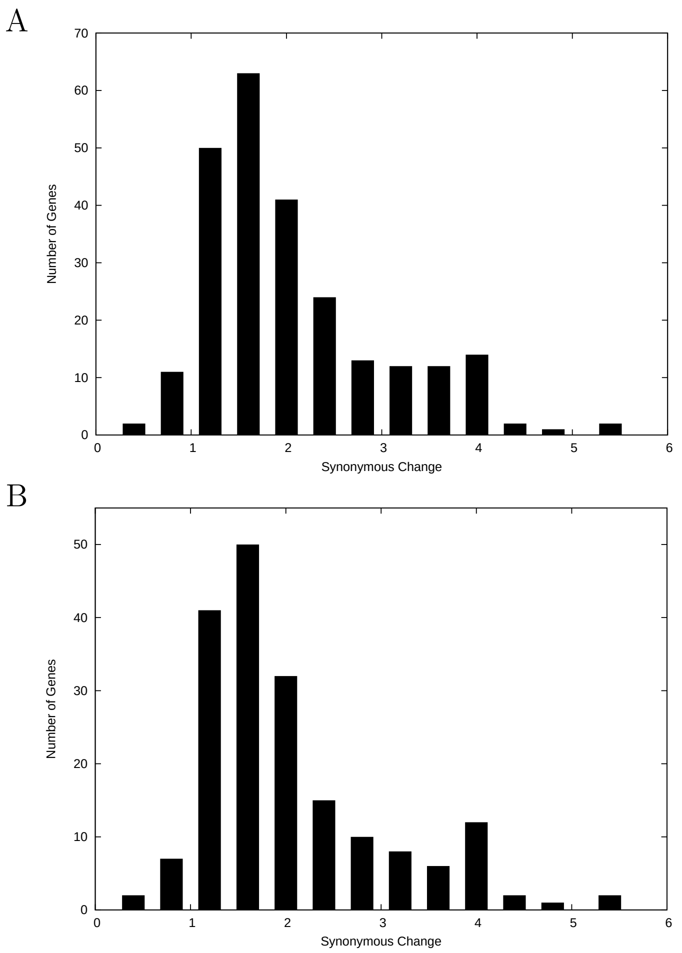

Figure S.2: Synonymous change of the genes present in Cgl and Cef. A, genes present in Cgl and Cef; B, genes present in Cgl and Cef and having matches in the database. Genes show similar rates of evolution by only looking at the genes having matches elsewhere the genome database.
