# Supplementary material for: The role of laterally transferred genes in adaptive evolution
Source: BMC Evol Biol. 2007 Feb 8;7(Suppl 1):S8. doi: 10.1186/1471-2148-7-S1-S8 (PMC1796617; doi:10.1186/1471-2148-7-S1-S8)
Supplement: Additional File 1 — The phyletic patterns of the Corynebacterium taxa. (cut-off: expect value less than 10-20 and match length over 85%) [file 1471-2148-7-S1-S8-S1.pdf]

Supplementary Information

Table S.1 - The phyletic patterns of the Corynebacterium taxa

Number of genes	Cje	Cdi	Cgl1	Cgl2	Cet
1141	1	1	1	1	1
835	0	0	1	1	0
537	0	0	1	1	1
442	0	0	0	0	1
417	1	0	0	0	0
299	0	1	1	1	1
281	0	1	0	0	0
126	1	0	1	1	1
64	1	1	0	0	0
54	1	0	0	0	1
51	0	1	0	0	1
40	0	1	1	1	0
31	1	0	1	1	0
30	1	1	1	1	0
17	1	1	0	0	1
11	0	0	0	1	0
5	0	0	1	0	0
1	1	1	0	1	1
4382	Total				
