# Supplementary material for: The role of laterally transferred genes in adaptive evolution
Source: BMC Evol Biol. 2007 Feb 8;7(Suppl 1):S8. doi: 10.1186/1471-2148-7-S1-S8 (PMC1796617; doi:10.1186/1471-2148-7-S1-S8)
Supplement: Additional File 2 — The phyletic patterns of the Corynebacterium taxa (cut-off: expect value less than 10-10 and match length over 70%) [file 1471-2148-7-S1-S8-S2.pdf]

Table S.2 - The phyletic patterns of the Corynebacterium taxa (cut-off: expect value less than 10^{-10} and match length over 70%)

Number of genes	Cje	Cdi	Cgl1	Cgl2	Cet
1129	1	1	1	1	1
673	0	0	1	1	0
397	0	0	1	1	1
261	0	0	0	0	1
248	0	1	1	1	1
239	1	0	0	0	0
185	0	1	0	0	0
112	1	0	1	1	1
53	1	1	0	0	0
39	0	1	0	0	1
37	1	0	0	0	1
31	1	1	1	1	0
30	0	1	1	1	0
25	1	0	1	1	0
21	1	1	0	0	1
5	0	0	1	0	0
1	1	1	0	1	1
1	0	0	1	0	1
1	0	0	0	1	0
3488	Total				
