# Supplementary material for: The role of laterally transferred genes in adaptive evolution
Source: BMC Evol Biol. 2007 Feb 8;7(Suppl 1):S8. doi: 10.1186/1471-2148-7-S1-S8 (PMC1796617; doi:10.1186/1471-2148-7-S1-S8)
Supplement: Additional File 3 — The phyletic patterns of the Corynebacterium taxa (cut-off: expect value less than 10-05 and match length over 50%) [file 1471-2148-7-S1-S8-S3.pdf]

Table S.3 - The phyletic patterns of the Corynebacterium taxa (cut-off: expect value less than 10^{-05} and match length over 50%)

Number of genes	Cje	Cdi	Cgl1	Cgl2	Cet
1067	1	1	1	1	1
583	0	0	1	1	0
297	0	0	1	1	1
183	0	1	1	1	1
172	0	0	0	0	1
155	1	0	0	0	0
135	0	1	0	0	0
96	1	0	1	1	1
44	1	1	0	0	0
36	1	0	0	0	1
30	0	1	1	1	0
23	1	0	1	1	0
22	1	1	1	1	0
22	1	0	0	0	1
17	1	1	0	0	1
2	1	1	0	1	0
2	0	0	1	0	0
1	1	0	1	0	0
1	1	0	0	1	1
1	0	1	0	1	0
1	0	0	1	0	1
1	0	0	0	1	0
2891	Total				
