# Supplementary material for: The role of laterally transferred genes in adaptive evolution
Source: BMC Evol Biol. 2007 Feb 8;7(Suppl 1):S8. doi: 10.1186/1471-2148-7-S1-S8 (PMC1796617; doi:10.1186/1471-2148-7-S1-S8)
Supplement: Additional File 4 — Insertion/deletion rates inferred from the maximum likelihood analysis assuming different rates for external and internal branches (cut-off: expect value less than 10-10 and > 70% match length) [file 1471-2148-7-S1-S8-S4.pdf]

Table S.4 - Insertion/deletion rates inferred from the maximum likelihood analysis assuming different rates for external and internal branches (cut-off: expect value less than 10^{-10} and $> 70\%$ match length)

Rate	Reversible		Deleted once[a]	
	MLE	LnL	MLE	LnL
constant μ	0.97	-8257.3	0.93	-8329.8
μ_1=μ_2=μ_3=μ_4=μ_5=μ_6 (external)	1.04	-8254.8	1.09	-8309.9
$\mu_7 = \mu_8$ (internal)	0.83		0.57	

[a]Genes can not be regained after deletion.
