# Supplementary material for: The role of laterally transferred genes in adaptive evolution
Source: BMC Evol Biol. 2007 Feb 8;7(Suppl 1):S8. doi: 10.1186/1471-2148-7-S1-S8 (PMC1796617; doi:10.1186/1471-2148-7-S1-S8)
Supplement: Additional File 5 — Insertion/deletion rates inferred from the maximum likelihood analysis assuming different rates on each branch of the phylogeny (cut-off: expect value less than 10-10 and > 70% match length) [file 1471-2148-7-S1-S8-S5.pdf]

Table S.5 - Insertion/deletion rates inferred from the maximum likelihood analysis assuming different rates on each branch of the phylogeny (cut-off: expect value less than 10^{-10} and $> 70\%$ match length)

Rate	Reversible		Deleted once[a]	
	MLE	LnL	MLE	LnL
constant μ	0.97	-8257.3	0.93	-8329.8
μ_1	1.02	-7981.3	0.96	-8014.5
μ_2	0.46		0.46	
μ_3	4.60		2.96	
μ_4	13.85		20.15	
μ_5	0.66		0.71	
μ_6	2.13		2.00	
μ_7	1.95		1.75	
μ_8	0.001		0.01	

[a]Genes can not be regained after deletion.
