# Supplementary material for: The role of laterally transferred genes in adaptive evolution
Source: BMC Evol Biol. 2007 Feb 8;7(Suppl 1):S8. doi: 10.1186/1471-2148-7-S1-S8 (PMC1796617; doi:10.1186/1471-2148-7-S1-S8)
Supplement: Additional File 7 — Insertion/deletion rates inferred from the maximum likelihood analysis assuming different rates on each branch of the phylogeny (cut-off: expect value less than 10-05 and > 50% match length) [file 1471-2148-7-S1-S8-S7.pdf]

Table S.7 - Insertion/deletion rates inferred from the maximum likelihood analysis assuming different rates on each branch of the phylogeny (cut-off: expect value less than 10^{-05} and $> 50\%$ match length)

Rate	Reversible		Deleted once[a]	
	MLE	LnL	MLE	LnL
constant μ	0.84	-6648.9	0.81	-6700.0
μ_1	0.80	-6356.3	0.79	-6384.8
μ_2	0.43		0.44	
μ_3	4.6		2.29	
μ_4	13.85		27.00	
μ_5	0.45		0.53	
μ_6	2.13		2.00	
μ_7	1.60		1.44	
μ_8	0.001		0.01	

[a]Genes can not be regained after deletion.
