# Supplementary material for: The role of laterally transferred genes in adaptive evolution
Source: BMC Evol Biol. 2007 Feb 8;7(Suppl 1):S8. doi: 10.1186/1471-2148-7-S1-S8 (PMC1796617; doi:10.1186/1471-2148-7-S1-S8)
Supplement: Additional File 13 — An alternative topology of the concatenated genes. The insertion/deletion rates on external branches and internal branches were studied. [file 1471-2148-7-S1-S8-S13.pdf]

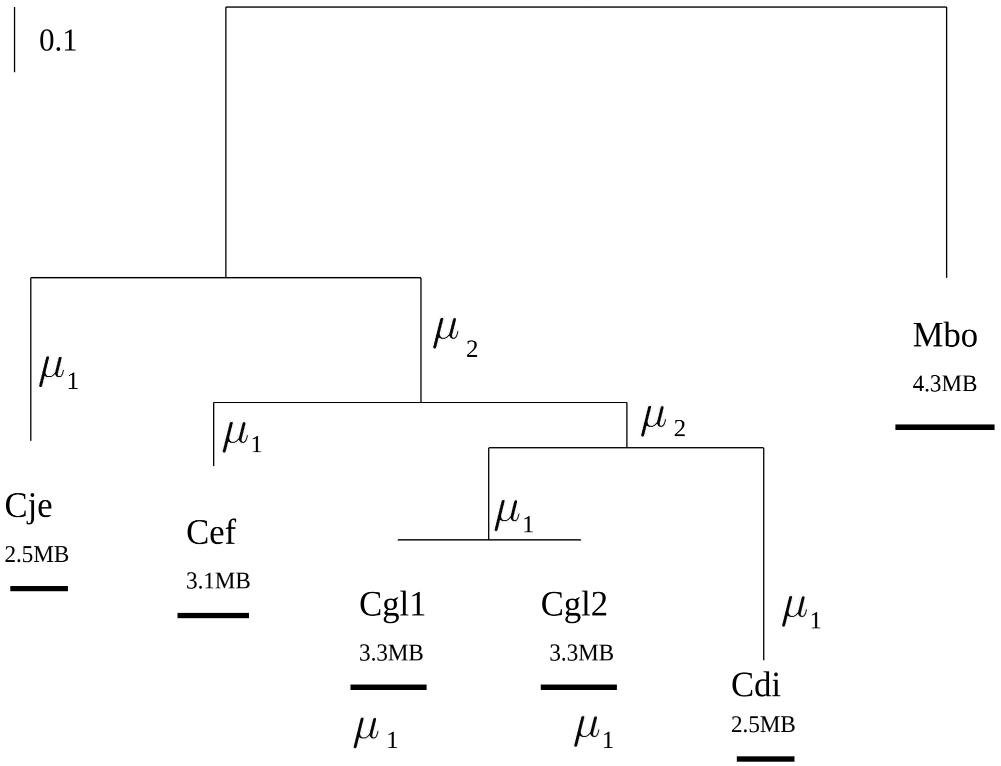

Figure S.4: An alternative topology of the concatenated genes. The insertion/deletion rates on external branches and internal branches were studied.
