# Supplementary material for: The role of laterally transferred genes in adaptive evolution
Source: BMC Evol Biol. 2007 Feb 8;7(Suppl 1):S8. doi: 10.1186/1471-2148-7-S1-S8 (PMC1796617; doi:10.1186/1471-2148-7-S1-S8)
Supplement: Additional File 8 — Insertion/deletion rates inferred from the maximum likelihood analysis assuming different rates for external and internal branches. Likelihood was estimated using the alternative topology and a cutoff with expect value less than 10-20 and > 85% match length. [file 1471-2148-7-S1-S8-S8.pdf]

Table S.8 - Insertion/deletion rates inferred from the maximum likelihood analysis assuming different rates for external and internal branches. Likelihood was estimated using the alternative topology and a cutoff with expect value less than 10^{-20} and $> 85\%$ match length.

Rate	Reversible		Deleted once[a]	
	MLE	LnL	MLE	LnL
constant μ	1.13	-11114.8	1.03	-11280.5
μ_1 (external)	1.32	-11067.0	1.39	-11069.6
μ_2 (internal)	0.15		0.004	

[a]Genes can not be regained after deletion.
