# Supplementary figures and images for: The role of laterally transferred genes in adaptive evolution
Source: BMC Evol Biol. 2007 Feb 8;7(Suppl 1):S8. doi: 10.1186/1471-2148-7-S1-S8 (PMC1796617; doi:10.1186/1471-2148-7-S1-S8)

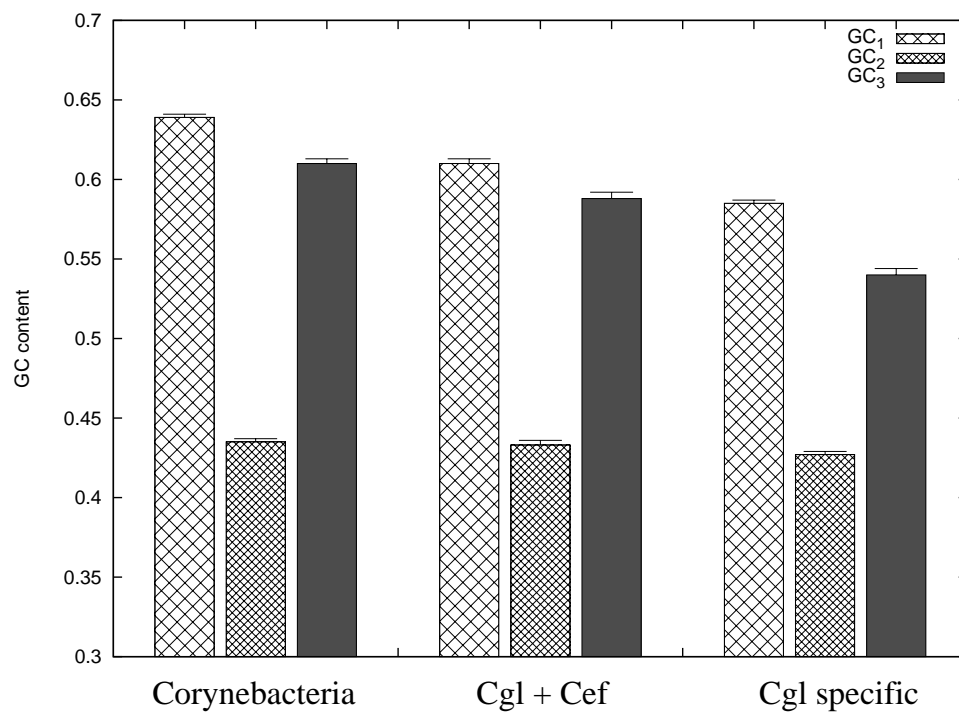

Figure S.7: Decreased GC content of recently acquired genes.

Supplement: Additional File 16 — Decreased GC content of recently acquired genes. [file 1471-2148-7-S1-S8-S16.pdf]
